# Soziale Arbeit am Limit? Professionsbezogene Folgen veränderter Arbeitsbedingungen in der Corona-Pandemie

**DOI:** 10.1007/s12054-021-00380-0

**Published:** 2021-04-22

**Authors:** Nikolaus Meyer, Elke Alsago

**Affiliations:** 1 Hochschule Fulda, Fulda, Deutschland; 2 Berlin, Deutschland

**Keywords:** Corona-Pandemie, Soziale Arbeit, Arbeitsbedingungen, Professionalität, Professionalisierung

## Abstract

Sozialer Arbeit kommt in der Corona-Pandemie eine mehrdimensionale Schlüsselfunktion bei der Bewältigung der Folgen ebendieser auf den Ebenen der Adressat_innen und der Gesellschaft zu. Gleichzeitig ist auch die Soziale Arbeit auf verschiedene Weisen von den Folgen der Pandemie betroffen. Der Beitrag thematisiert die Situation der Beschäftigten aus unterschiedlichen Handlungsfeldern und zeigt, dass sich die Arbeitsbedingungen in der Sozialen Arbeit während der Corona-Pandemie weiter verschlechtern.

Der „Kampf um Anerkennung“ begleitet die historische Entwicklung der Sozialen Arbeit. Eine Online-Befragung unter über 3000 Beschäftigten während des zweiten Lockdowns zeigt: Sowohl Professionalisierung als auch Professionalität geraten unter Veränderungsdruck.

Das Auftreten des Virus SARS CoV‑2 Ende 2019 und die von ihm ausgelöste Lungenerkrankung fungiert als Brennglas, in dem sich durch sie bereits bestehende Probleme und Disparitäten verschärfen: Menschen waren und sind sowohl von der Erkrankung als auch von deren Folgen völlig ungleich betroffen (vgl. Beuchat und Grob 2020; Huang 2020). Zudem bewirken die Corona-Pandemie und die mit ihr verbundenen Schutzmaßnahmen bisher unbekannte wirtschaftliche, psychische und soziale Veränderungen (vgl. DIW 2020; DBSH 2020), die aktuell weder mit Blick auf konkrete gesellschaftliche noch auf individuelle Veränderungen abschließend beurteilt werden können (vgl. Meyer und Buschle 2021a).

Der Sozialen Arbeit kommt in dieser komplexen und unabgeschlossenen Gemengelage im Prozess der Bewältigung der Krise und ihrer Folgen eine mehrdimensionale „Schlüsselfunktion“ (IFSW 2020) zu.^1^ Gleichzeitig ist Soziale Arbeit selbst von der Pandemie berührt. Dies zeigt sich in ersten Studien sowohl bei den Beschäftigten^2^ (Meyer und Buschle 2020) als auch bei den Organisationen (BFS 2020). Vor dem Hintergrund dieser vielfältigen Aufgabenstellungen für die Beschäftigten in der Sozialen Arbeit stellt der vorliegende Beitrag erste Tendenzen einer Onlinebefragung von 3064 Berufstätigen aus unterschiedlichen Handlungsfeldern der Sozialen Arbeit (vgl. Meyer und Siewert 2021) vor, die zwischen dem 9. November und dem 6. Dezember 2020 – also während des zweiten Lockdowns – stattfand.

Mit den in der Befragung erhobenen Daten arbeiten wir in diesem Beitrag die aktuelle Beschäftigungssituation in der Sozialen Arbeit heraus und benennen exemplarisch Unterschiede zwischen ausgewählten Handlungsfeldern. Den Beitrag schließen wir mit einem Ausblick auf mögliche langfristige Folgen für die Soziale Arbeit durch die Corona-Pandemie, wobei eine abschließende Beurteilung angesichts der offenen Gesamtsituation derzeit unmöglich ist. Vielmehr werden aktuelle Gefahren für die Professionalisierung – wir verstehen hier den kollektiven Prozess „der Aufwertung und Institutionalisierung einer spezifischen Form von Beruflichkeit im Strom der Zeit“ (Nittel 2011, S. 44) – Sozialer Arbeit beschrieben.

## Die Arbeitssituation im zweiten Lockdown

Die Teilnehmenden (*n* = 3064) an der nicht-repräsentativen Befragung während des zweiten Lockdowns kommen aus den unterschiedlichsten Handlungsfeldern der Sozialen Arbeit, vor allem aus den Bereichen Kinder- und Jugendhilfe (28,3 %), Elementarbildung (23,6 %), Soziale Arbeit mit Menschen mit Beeinträchtigung (7,9 %), Soziale Arbeit in Schulen (7,4 %), Soziale Arbeit im Gesundheitswesen (4,9 %), Soziale Arbeit im Kontext von Migration^3^ (2,6 %), Soziale Arbeit mit Menschen in prekären Lebenslagen (2,3 %) oder Arbeitslosen (2,3 %) sowie Soziale Arbeit im Kontext von Flucht und Asyl^4^ (2,2 %).

## Institutionelle Rahmenbedingungen

**Figure 1:**
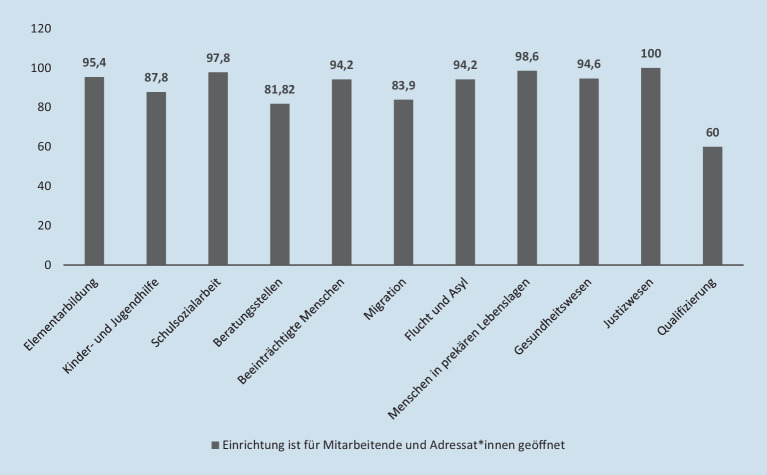

Zum Zeitpunkt der Befragung waren Einrichtungen der Sozialen Arbeit – trotz des *Lockdown lights* ab dem 28. Oktober und der Verschärfung ab dem 25. November 2021 – tendenziell eher geöffnet. So war bei 89,8 % der Beschäftigten die Einrichtung für Mitarbeitende wie Adressat_innen geöffnet und lediglich bei 7,3 % der Teilnehmenden war nur Mitarbeitenden der Zutritt erlaubt. Vollständig geschlossen waren die Einrichtungen über alle Handlungsfelder hinweg nur bei 0,7 % der Befragten, was sich auch in der niedrigen Quote von Kurzarbeitenden (7,2 %) ausdrückt. Allerdings waren die Öffnungsquoten zwischen den Handlungsfeldern sehr unterschiedlich verteilt (Abb. 1).

**Figure 2:**
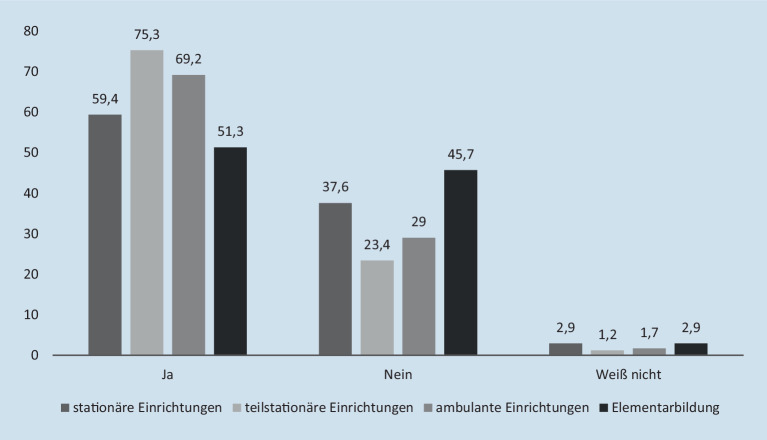

Erschließt man die Öffnung von Einrichtungen nicht nur über die Handlungsfelder, sondern auch über den Modus ihrer Organisation – also ambulant, teilstationär oder stationär (Mund 2019) – ergibt sich ein noch differenzierteres Bild: Hier sind stationär-arbeitende Einrichtungen besonders häufig für Mitarbeitende und Adressat_innen geöffnet (96,3 %), wohingegen teilstationäre (89,5 %) oder ambulante (85,7 %) Einrichtungen eine leicht höhere Schließungsquote aufweisen. Dabei sind alle Einrichtungen unabhängig vom Organisationsmodus Begrenzungen im Hinblick auf die Anzahl möglicher Angebote unterworfen (Abb. 2). Gleichwohl sind aus Sicht der befragten Beschäftigten die teilstationären Angebote mit Ausnahme der Elementarbildung – dieser Bereich weist mit die geringsten Angebotsbegrenzungen auf (45,7 %) – die am stärksten von solchen Beschränkungen betroffen Handlungsformen (75,3 %).

Insgesamt fällt in dieser Betrachtung der vorliegenden Daten auf, dass die Befragten aus stationären Einrichtungen der Sozialen Arbeit (59,4 %) sowie der Elementarbildung die geringsten Angebotsbegrenzungen wahrnehmen.

Verbleibt man auf der Ebene der Organisationsform der Einrichtungen, zeigt sich, dass sich in der Wahrnehmung der Beschäftigten, neben den dargestellten Angebotsbegrenzungen oder Schließungen, auch die Nachfrage nach Angeboten ungleich entwickelt: Während im teilstationären (27,3 %) sowie stationären Setting (26,4 %) die Beschäftigten eine gestiegene Nachfrage von bis zu einem Viertel wahrnehmen, sind es im ambulanten Bereich sogar 40,5 % der befragten Beschäftigten. Kehrt man an dieser Stelle auf die Differenzierungsebene der Handlungsfelder zurück, zeigen sich die wahrgenommenen Anstiege bei der Nachfrage vor allem bei der Sozialen Arbeit mit Menschen in prekären Lebenslagen (50 %), mit Frauen (50 %), der sozialraumbezogenen Arbeit (48,7 %), der Sozialen Arbeit im Bereich Migration (39,4 %) sowie der Tätigkeit mit alten Menschen (39,1 %). Gleichzeitig sind aus Sicht der Befragten einige Handlungsfelder dazu übergegangen, Hilfen früher als üblich zu beenden. Dies betrifft die Soziale Arbeit im Justiz- (25,9 %) sowie Gesundheitswesen (18,9 %) oder mit älteren Menschen (22,7 %) ebenso wie Beratungsstellen (33,3 %) oder die Arbeit mit Erwerbslosen (20,9 %).

Insgesamt nimmt die Anzahl der Adressat_innen pro beschäftigter Person zu (35,0 %), wobei die Elementarbildung (58,8 %) und die Soziale Arbeit mit beeinträchtigten Menschen (45,8 %) sowie die Beratungsstellen (45,4 %) die höchste Steigerung erfahren.

## Fehlende Steuerung bei Schutzmaßnahmen im Arbeitskontext

Die Arbeitsbelastung hat sich neben den beschriebenen Effekten allerdings auch durch die bereits vor der Corona-Pandemie angespannte Personalsituation im Zuge des Fachkräftemangels (Fischer und Graßhoff 2020) in den Einrichtungen weiter verschärft: Insgesamt wurden bei 11,1 % der Befragten, erwartungsgemäß vor allem den lebensälteren Personen, ein ärztlich festgestelltes – zumeist durch die Hausärzt_innen (86 %) – höheres Risiko für einen schweren Verlauf einer COVID 19-Erkankung attestiert. Die betroffenen Beschäftigten sind im Arbeitskontext jedoch weitgehend ohne Unterstützung geblieben. So geben 66,3 % der Befragten an, dass man individuell oder im Team Maßnahmen zum Eigenschutz ergriffen habe. 18,4 % der Befragten müssen sogar ohne zusätzliche Schutzmaßnahmen arbeiten, weil der/die jeweilige Arbeitgeber_in ihnen keine andere Wahl lasse.

Diese fehlende Steuerung durch Leitungskräfte zeigt sich auch mit Blick auf die Frage, wer auf die Einhaltung der Schutzmaßnahmen in der eigenen Einrichtung oder Organisation achtet. Hier wählen über alle Handlungsfelder hinweg 46,4 % der Befragten die Antwortoption, dass sich die Vorgesetzten nicht um die Einhaltung der Schutzmaßnahmen bemühten. Vielmehr haben sich die Beschäftigten in der Sozialen Arbeit gemeinsam im Team organisiert, um die Einhaltung der Schutzmaßnahmen zu überprüfen (80,3 %). Auch die betriebliche Interessenvertretung spielte dabei aus der Perspektive der Beschäftigten nur eine geringe Rolle (10,7 %).

Während zwischen den Handlungsfeldern recht unterschiedliche Verteilungen bei der Rolle der verantwortlichen Person oder Gruppe sichtbar sind, ist dies auf der Ebene der Trägerschaft nicht nachweisbar. Hier liegen öffentlicher Dienst, privatwirtschaftlich organisierte und freigemeinnützige Träger eng beieinander.

Einfluss auf die Wahrnehmung der Arbeitsbedingungen in der Sozialen Arbeit haben, neben der Frage nach Öffnungen, möglichen Beschränkungen und veränderter Nachfrage, allerdings auch die Möglichkeiten zum konkreten Schutz gegenüber den Coronaviren: Im Vergleich zu den Erkenntnissen aus dem ersten Lockdown (Buschle und Meyer 2020; Meyer und Buschle 2020) veränderte sich im zweiten Lockdown die Lage im Bereich der Schutzausrüstung (Mund-Nasen-Schutz, Desinfektionsmittel etc.) in der Sozialen Arbeit insgesamt positiv.^5^ Im November und Dezember 2020 bestätigten die Beschäftigten weitgehend, dass zwischenzeitlich Schutzausrüstung in angemessenem Maß in den Einrichtungen vorhanden ist (78,4 %), sodass der Betrieb nicht offiziell eingeschränkt werden muss. Gleichzeitig gaben allerdings fast 13 % der Befragten an, dass es aufgrund fehlender Schutzausrüstungen bei ihnen in der Einrichtung zu Schließungen gekommen sei. Auch dieser Mangel ist zwischen den Handlungsfeldern unterschiedlich verteilt: Die beste Versorgung mit Schutzausrüstungen wurde im Bereich der Sozialen Arbeit mit älteren Menschen (92 %) sowie in der Betriebssozialarbeit (100 %) realisiert. Die Trägerart hat in der Frage nach der angemessenen Ausstattung mit Schutzausrüstung insgesamt nur geringen Einfluss: Die Beschaffung von ausreichend Schutzausrüstung zur Verhinderung einer Angebotsreduktion ist dabei in privatwirtschaftlichen (75,4 %) und öffentlichen (73,2 %) Institutionen geringer ausgeprägt als im frei-gemeinnützigen (87,3 %) oder kirchlichen (82,1 %) Bereich.

**Figure 3:**
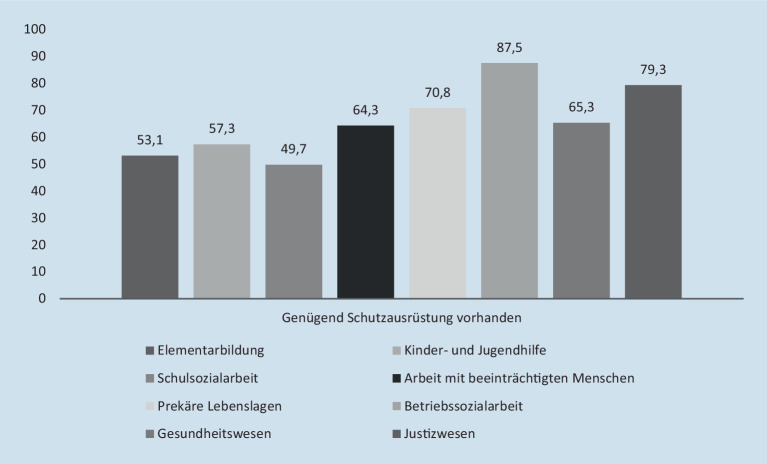

Die Befragten verweisen allerdings auch darauf, dass zwar genügend Schutzausrüstung zur Aufrechterhaltung des Betriebs vorhanden sei, es aber weiterer Anstrengungen bedürfe, um Mitarbeitende wie Adressat_innen wirklich dauerhaft, umfassend und effektiv schützen zu können – hier sind über alle Handlungsfelder Sozialer Arbeit hinweg nur 60,4 % der befragten Beschäftigten mit den vorhandenen Möglichkeiten zufrieden. Auch diese Perspektive differiert zwischen den Handlungsfeldern (Abb. 3).

Waren in den Fragen zuvor mit Schutzausrüstung auch Materialen wie Desinfektionsmittel gemeint, zeigt sich beim Einsatz persönlicher Schutzausrüstung im Alltag der Befragten eine deutliche Veränderung: Über 15 % der Befragten tragen keine solche persönliche Schutzausrüstung wie FFP2-Masken o. ä.. Aus den quantitativen Daten lassen sich die Gründe nicht eindeutig ableiten: So sind fehlende Schutzausrüstungen ebenso wie pädagogische Erwägungen denkbar. Diese These scheint angesichts der am stärksten betroffenen Handlungsfelder nicht unwahrscheinlich und wird durch Aussagen im offenen Teil des Fragebogens gestützt: So sind vor allem die Elementarbildung (32,1 %) sowie in geringerem Maße die Kinder- und Jugendhilfe (18,1 %) sowie die Arbeit mit Frauen (15,7 %) betroffen. Gerade in diesen Handlungsfeldern sowie in der Sozialen Arbeit mit beeinträchtigten oder älteren Menschen berichteten die Befragten gleichzeitig besonders häufig über eine empfundene Unmöglichkeit der Einhaltung des Sicherheitsabstands von 1,5 Metern. Anders ausgedrückt: Just in den Handlungsfeldern, in denen die Beschäftigten am seltensten persönliche Schutzausrüstung im Alltag tragen, ist es am schwierigsten, sich durch Abstand halten im Dienst zu schützen. Ergebnisse zu der im Vergleich mit anderen Berufsgruppen hohen Erkrankungsrate der Beschäftigten in Erziehungs- und Sozialdienst überraschen hier nicht (Barmer 2021; WIdO 2020). Bemerkenswert ist der Befund, dass die Beschäftigten selten Kenntnis davon hatten, ob ihre Arbeitgeber die gesetzlich vorgesehenen Gefährdungsbeurteilungen^6^ durchgeführt hatten (68,6 %). Vielmehr ist bei der zahlreichen Nutzung der Antwortoption „Weiß nicht“ zu vermuten, dass dieses arbeits- und gesundheitsschutzrechtliche Instrumentarium sowie die damit verbundene Mitwirkung durch die Personalvertretungen vielfach unbekannt zu sein scheint.

## Veränderte Interaktion mit Adressat_innen

**Figure 4:**
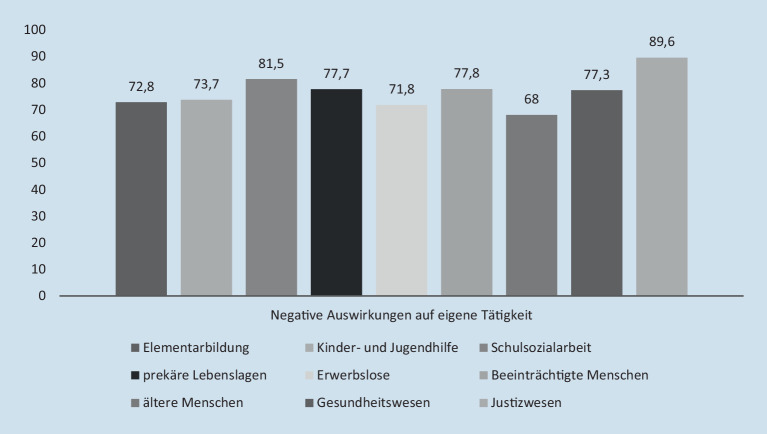

Trotz der skizzierten eigenen Ansteckungsrisiken stoßen bei den Befragten die angeordneten staatlichen Schutzmaßnahmen und trägereigene Vorgaben auch auf erhebliches Unverständnis, weil sie aus deren Sicht dem Berufswissen und gängigen Standards widersprechen. Die Beschäftigten geben über alle Handlungsfelder hinweg auch an (74,4 %), dass die getroffenen Schutzmaßnahmen die eigene Arbeit negativ veränderten (Abb. 4).

In den offenen Antworten (*n* = 2404) fokussieren die Befragten auf den Umstand, dass durch die Maßnahmen Adressat_innen verunsichert oder überhaupt nicht mehr erreicht würden. Hier setzen sich negative Einschätzungen der Befragten zu den Folgen für die Arbeit aus der ersten Erhebungswelle im Frühjahr fort (Meyer und Buschle 2021b). Die These der verunsicherten Adressat_innen wird im quantitativen Teil gestützt: Hier geben 38,6 % der Befragten an, dass seit Ausbruch der Corona-Pandemie die Adressat_innen häufig oder sehr häufig Termine absagen – besonders häufig in der Kinder- und Jugendhilfe mit 46,8 %. Dabei haben aus Sicht von gut drei Viertel der befragten Beschäftigten die Problemlagen der Adressat_innen während der Corona-Pandemie deutlich zugenommen (71,5 %) und jede zweite Person geht darüber hinaus von einem gestiegenen Armutsrisiko bei den Adressat_innen aus (57,2 %).^7^

**Figure 5:**
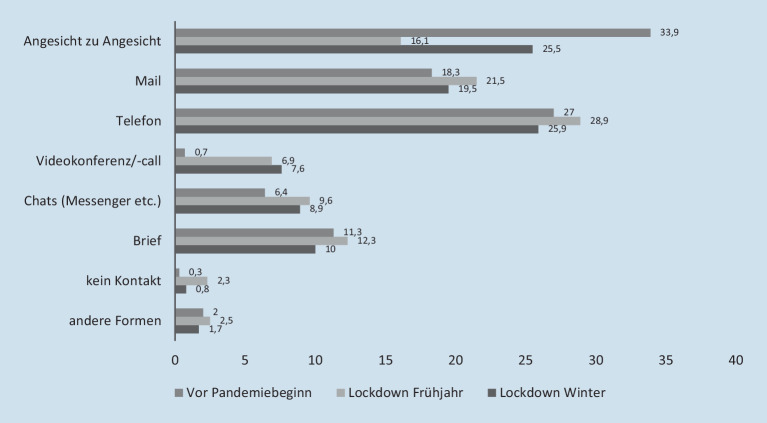

Ähnlich negative Einschätzungen zeigen sich über alle Handlungsfelder hinweg im Modus der Kontaktaufnahme mit den Adressat_innen. So wählen rund 95 % der Befragten den Kontaktmodus *Angesicht zu Angesicht* und geben in einer weiteren Frage an, dass dieser Anteil seit Ausbruch der Corona-Pandemie gesunken sei (78,6 %). Setzt man die einzelnen Handlungsmodi in ein Verhältnis zueinander, erhält man Auskunft über den Anteil der verschiedenen Kontaktformen innerhalb des Arbeitsalltags über alle Handlungsfelder Sozialer Arbeit hinweg (Abb. 5).

Insgesamt wird hier eine leichte Verschiebung deutlich: Waren die befragten Beschäftigten während des ersten Lockdowns, auch aufgrund von Schließungen, zu einer stark veränderten Kommunikation gezwungen, hat der Kontakt Angesicht zu Angesicht zu den Adressat_innen danach wieder zugenommen, auch weil bei 40,4 % der Befragten die Adressat_innen über keine ausreichende Möglichkeit zur digitalen Kontaktaufnahme verfügen.^8^ Diese spezifische Form der direkten Interaktion mit den Adressat_innen wird dabei von den Beschäftigten im öffentlichen Dienst am seltensten gewählt und nimmt entsprechend einen geringeren Anteil innerhalb des Sets der verschiedenen Kontaktmodi ein (23,8 %). Parallel zeigen sich auch hier Unterschiede zwischen den Handlungsfeldern. Über alle Handlungsfelder hinweg ist gleichzeitig die Quote derjenigen gesunken, die zu ihren Adressat_innen keinen Kontakt mehr haben. Dennoch zeigt sich hier eine Verschiebung im professionellen Handeln: Insgesamt sind die Handlungsmodi digitaler geworden und weniger im Bereich persönlicher Kontakte zu verorten. Parallel hat sich aus Sicht der Beschäftigten die Anzahl der Kontakte mit den Adressat_innen auch im zweiten Lockdown weiter verringert^9^: So gibt über alle Handlungsfelder hinweg mehr als jede zweite befragte Person eine solche Entwicklung an (53,9 %), wobei diese in der Elementarbildung (54,5 %), der Kinder- und Jugendhilfe (55,1 %) sowie der Arbeit mit Menschen in prekären Lebenslagen (60 %) am stärksten ausgeprägt ist.

Im Ergebnis haben sich aus Sicht der Beschäftigten die problematischen Handlungsmuster in der Lebensbewältigung der Adressat_innen verstärkt (63 %), wobei dies besonders stark in der Sozialen Arbeit mit Frauen (83,3 %) oder Menschen in prekären Lebenslagen (81,2 %) zugenommen hat sowie in der Arbeit mit Erwerbslosen (74,2 %) oder der Schulsozialarbeit (72,8 %).

## Veränderte Arbeitsumwelt: Homeoffice und Kooperation

**Figure 6:**
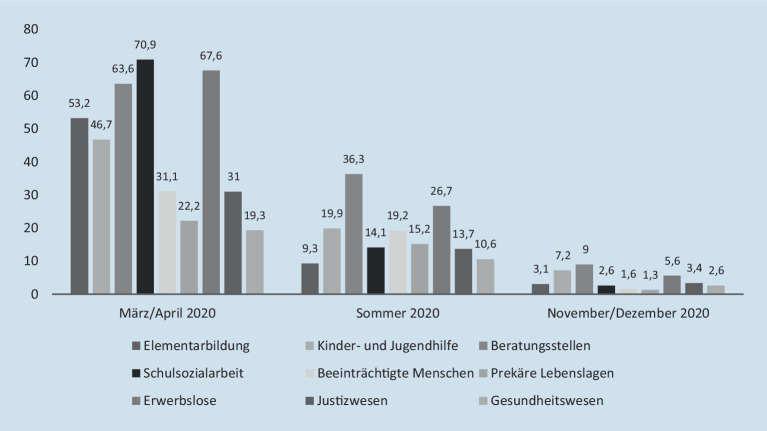

Ebenfalls von hoher Bedeutung für die Veränderung der Arbeitsweisen war auch im zweiten Lockdown das Homeoffice (Abb. 6): Arbeiteten im ersten Lockdown noch rund 60 % der Teilnehmenden zum Befragungszeitpunkt von zu Hause aus, waren im November und Dezember 2020 nur 6,9 % der Befragten fest im Homeoffice tätig, 26,1 % ab und zu sowie 66,7 % gar nicht (Meyer et al. 2021). Die Möglichkeit, Homeoffice zu nutzen, besaßen zumeist Leitungskräfte und Beschäftigte im öffentlichen Dienst. Nur knapp jede zweite Person im Homeoffice erhielt dabei vom jeweiligen Arbeitgeber die notwendige Ausstattung wie PC, Papier oder Bildschirm (49,2 %), wobei die Ausstattung häufiger bei frei-gemeinnützigen (58,7 %) und kirchlichen Trägern (56,7 %) sowie seltener im öffentlichen Dienst (39,6 %) sowie bei privatwirtschaftlich organisierten Trägern (47 %) gestellt wurde.

Insgesamt bewerten jene Befragte, die mit der Arbeit im Homeoffice zufrieden sind, dies auch als eine grundsätzlich positive Veränderung ihrer Arbeitsbedingungen und erkennen in den Veränderungen ihrer Arbeitsbedingungen positive Anteile, die sie nach Ende der Corona-Pandemie beibehalten möchten.

Parallel geben die Befragten an, dass sich während der Corona-Pandemie sowohl der Kontakt zu den Vorgesetzten (55,3 %), den Kolleg_innen (75,4 %) und den Kooperationspartner_innen (76,5 %) verändert und sich im Ergebnis auch die eigene Arbeit verschlechtert hat. Gerade der veränderte Austausch mit den Kolleg_innen zeigt für die Befragten dabei besonders negative Dimensionen (54,5 %), die in den offenen Antworten an unterschiedlichen Aspekten gezeigt werden: Von täglich wechselnden Teamzusammenstellungen bis hin zur Absenkung von Standards, wie etwa der Reduzierung der notwendigen Anzahl der Mitglieder für ein Fachteam im Allgemeinen Sozialen Dienst des Jugendamts.

## Blick in die Zukunft

Nach der Corona-Pandemie rechnen die Befragten der zweiten Befragungswelle wie auch bereits jene der ersten (Buschle und Meyer 2020) weiter mit zunehmenden Anforderungen an das eigene Arbeitsfeld. Dabei glauben 46,7 %, dass die Belastung des jeweils eigenen Handlungsfeldes zukünftig steigt, wohingegen 44,8 % von einer gleichbleibenden Belastung ausgehen. Während die Beschäftigten in den Handlungsfeldern der Kinder- und Jugendhilfe, der Arbeit mit Menschen in prekären Lebenslagen sowie mit Erwerbslosen und der Sozialen Arbeit in Schulen mehrheitlich von sich verschärfenden Bedingungen für die Zukunft ausgehen, rechnen die Angestellten in der Elementarbildung, der Sozialen Arbeit im Gesundheitsbereich und in der Arbeit mit Menschen mit Beeinträchtigung tendenziell mit dem Ausbleiben von Veränderungen für das eigene Handlungsfeld (Meyer et al. 2021).

## Soziale Arbeit in der Pandemie: Professionsbezogene Folgen

Die beschriebenen Veränderungen haben auf die Professionalität von Sozialarbeitenden in unterschiedlichen Dimensionen erheblichen Einfluss. Auf dieser Handlungsebene (Nittel 2011) sind die Beschäftigten in der Sozialen Arbeit in der Pandemie zunächst mit einer neuen Paradoxie professionellen Handelns konfrontiert. Sie müssen im Alltag zwischen von außen kommenden arbeitsschutz- wie infektionsschutzrechtlichen Maßgaben, die oft nicht eingehalten werden, und den berufsgruppenimmanenten Wissensbeständen changieren. Anders ausgedrückt: Die Beschäftigten in der Sozialen Arbeit stehen aktuell jeden Tag vor der Frage, wieviel Schutz bei wieviel Aufrechterhaltung professioneller Standards möglich ist. Hier sollten politische Entscheidungen künftig mit Vertreter_innen der Sozialen Arbeit vorab beraten werden, denn einerseits hat die Soziale Arbeit in der Pandemie eine zentrale gesellschaftliche Funktion (IFSW 2020) und verfügt andererseits über relevante Wissensbestände zur professionellen Gestaltung der komplexen Tätigkeit mit den Adressat_innen des jeweiligen Handlungsfeldes (Thole 2012). Dieses Wissen könnte für die Planung von Schutzmaßnahmen sinnvoll genutzt werden und damit gleichzeitig ein wichtiger Beitrag für die eigenwahrgenommene Arbeitszufriedenheit geleistet werden. Immerhin beschreiben zahlreiche Beschäftigte in den offenen Antworten der Befragung einen zunehmenden Druck im Zuge der pandemiebedingten Schutzmaßnahmen auf ihre Handlungen durch als unpassend erlebte Vorgaben von außen.

Gleichzeitig verändert sich auf dieser Handlungsebene das Arbeitsbündnis (Oevermann 1996) zwischen Beschäftigten und Adressat_innen – einem bedeutungsvollen Ort professionellen Handelns in der Sozialen Arbeit. So nehmen in der Wahrnehmung der Beschäftigten bei den Adressat_innen in der Pandemie vorhandene Problemlagen zu und gleichzeitig wachsen deren Armutsrisiken. Beides bewirkt eine Verschärfung der ohnehin oft prekären Lebensverhältnisse der Adressat_innen, was wiederum unmittelbaren Einfluss auf die professionelle Interaktion mit den Beschäftigten hat. Immerhin, darauf weisen Praktiker_innen aus unterschiedlichen Handlungsfeldern hin (Meyer und Siewert 2021), bedarf es im Zuge der Etablierung eines Arbeitsbündnisses zunächst einer Stabilisierung der Lebensverhältnisse, um Möglichkeiten der Adressierbarkeit zu schaffen. Gleichzeitig bringen Zuspitzungen der sozialen und/oder ökonomischen Bedingungen in der Lebenswelt der Adressat_innen auch in bereits etablierten Arbeitsbündnissen neue Krisen mit sich (Steinert und Ebert 2020; DGfE 2020; Müller 2020).

Parallel zu den negativen Veränderungen in der Lebenswelt der Adressat_innen vollzieht sich ein grundsätzlicher Wandel im Modus der Kontaktaufnahme zwischen ihnen und den Beschäftigten weg vom Kontaktmodus *Angesicht zu Angesicht*. Hier gilt es, die weitere Entwicklung in der Berufsgruppe im Blick zu behalten, denn die Arbeit mit Videokonferenztools – dies scheint zum Befragungszeitpunkt der stärkste Ersatz für persönlichen Kontakt – weist einen voraussetzungsvollen Charakter auf. Immerhin muss entsprechende IT bei den Adressat_innen vorhanden sein, was aus Sicht der befragten Beschäftigten allerdings für über 40 % ein Problem darstellt.

Daneben verändert sich nicht nur die Art der Interaktion zwischen Adressat_innen und Beschäftigten, sondern auch die Häufigkeit: Aus Sicht der Beschäftigten sagen Adressat_innen seit Ausbruch der Corona-Pandemie häufiger geplante Termine ab. Gleichzeitig verringern sich die quantitativen Möglichkeiten zur professionellen Intervention von Sozialarbeitenden. Zwar sind die Einrichtungen im zweiten Lockdown häufiger geöffnet und doch müssen die Beschäftigten durch die Beschränkungen bei den Angeboten mehr Veranstaltungen o. ä. möglich machen. Entsprechend geben die Beschäftigten an, dass sie im zweiten Lockdown mehr Adressat_innen parallel begleiten (24,8 %) müssen. Dies auch, weil eigene Kolleg_innen als Angehörige einer Risikogruppen ausfallen (18,3 %) oder selbst erkrankt sind (47,2 %).

Gleichzeitig werden Hilfen bei 13,3 % der Befragten früher als üblich beendet, vermutlich auch, um der gestiegenen Nachfrage zu begegnen. Damit verringert sich zwar der Wert aus dem ersten Lockdown^10^, bleibt allerdings auf hohem Niveau. Aber auch der veränderte Austausch mit Vorgesetzten, Kolleg_innen sowie Kooperationspartner_innen verschlechtert die eigenwahrgenommene Arbeitsqualität und evoziert Unzufriedenheit. Immerhin berichten Beschäftigte in den offenen Antworten noch immer über die Aussetzung von Fachteams zur Abwendung von Kindeswohlgefährdungen oder der Unerreichbarkeit von Vorgesetzten wie Kolleg_innen im Homeoffice. Daneben weisen die Beschäftigten in den offenen Antworten auch daraufhin, dass beispielsweise im Elementarbereich zunehmend fachfremde Personen parallel zur Bewältigung der pandemiebedingten Herausforderungen eingearbeitet werden müssen. Hier wurden in zahlreichen Bundesländern die Fachkraftregeln während des ersten Lockdowns gelockert (Buschle und Meyer 2020) und anschließend nicht wieder in ihren Regelzustand zurückversetzt.

Im Ergebnis verdichtet sich im Empfinden der Beschäftigten die eigene Arbeit einerseits und verändert sich andererseits nahezu vollständig (88,6 %). In dieser Gemengelage fühlen sich 62,1 % der Beschäftigten belastet oder sogar extrem. Insofern verschlechtern sich aus Sicht von jedem Zweiten die Arbeitsbedingungen und entsprechend denken 29,9 % über einen Stellen- sowie 16,2 % sogar über einen Berufswechsel nach.

Ist die Corona-Pandemie damit für die Soziale Arbeit nur negativ? Nein, es zeigen sich auch positive Facetten, wie beispielsweise in der Wahrnehmung der befragten Beschäftigten die zunehmenden Möglichkeiten des Homeoffice. Vor allem ist die hohe Selbstorganisation der Beschäftigten bedeutungsvoll (Meyer 2019): Im Team kümmern sie sich, auch wenn dies nicht ihre Aufgabe ist, um den eigenen Schutz oder haben trotz starker Beschränkungen innovative Formate auf den Weg gebracht. Diese Kraft der Selbstorganisation wird für den Prozess der weiteren Professionalisierung sicher bedeutsam sein – muss aber von Verbesserungen der Arbeitsbedingungen flankiert werden, für die es sich zu streiten lohnt.

## Footnotes

1 Trotz dieser bedeutsamen Stellung in der Bekämpfung der Pandemie wie ihrer Folgen bei gleichzeitig intensivierter Berichterstattung zum Themengebiet Corona-Pandemie (DLF 2021), wird Sozialer Arbeit im medialen Diskurs nur wenig Beachtung geschenkt (Meyer 2021). Im Gegenteil geben Angehörige anderer Berufsgruppen (z. B. Psycholog_innen, Kinderärzt_innen) in der Presse sogar über exklusive Tätigkeitsbereiche der Sozialen Arbeit (z. B. Kinderschutzverfahren, Bildungsprozesse) Auskunft. Aus professionstheoretischer Perspektive ist dies ebenso problematisch wie die Vielstimmigkeit im Wettbewerb um mediale Aufmerksamkeit während des ersten Lockdowns durch Fach- und Berufsverbände der Sozialen Arbeit (ebd.). Eine einheitliche Position zeigt sich hier nicht. An dieser Stelle ist grundsätzlich zu fragen, inwiefern diese Zersplitterung der Sozialen Arbeit *ihrer* kollektiven Professionalisierung dienlich ist.

2 Unter dem Begriff der *Beschäftigten* fassen wir die heterogenen Beschäftigungsbedingungen derjenigen Tätigen (z. B. verbeamtet, angestellt, freiberuflich) in der Sozialen Arbeit zusammen (Braches-Chyrek et al. 2021), die über eine Qualifizierung für die Soziale Arbeit verfügen. Dies schließt schulische wie hochschulische Qualifizierung ein, wobei diese nicht immer in den gesetzlichen Status der Fachkraft passen, der in der Sozialen Arbeit lediglich für das Handlungsfeld der Kinder- und Jugendhilfe im Sozialgesetzbuch (SGB) VIII gerahmt ist. So sind Sozialassistent_innen oder Kinderpfleger_innen im Sinne des SGB VIII keine Fachkräfte und haben doch eine individuelle Lizenzierung für die Tätigkeit in der Sozialen Arbeit durchlaufen. Diese augenscheinliche Differenz suchen wir mit dem Begriff der Beschäftigten zu umgehen, um damit die qualifizierte Hauptberuflichkeit zu betonen.

3 Hier subsumieren wir Tätigkeiten im Zusammenhang mit Integrationskursen, Ausländerrecht, Sozialberatung, Migrationsdienst, Frauenprojekten etc.

4 Hier subsumieren wir Tätigkeiten im Zusammenhang mit der Betreuung (ambulant bis stationär) sowie psychosozialen und freien Beratungsstellen für Geflüchtete.

5 „Mit Blick auf die adäquate und zum Teil in einigen Bundesländern rechtlich geforderte Schutzausrüstung für einige Bereiche zeigte die Befragung (n = 1607) [im ersten Lockdown, A. d. A.], dass diese in den Einrichtungen zumeist fehlte (70,07 %). Nur bei rund 14 % waren diese im ersten Lockdown in ausreichendem Maße vorhanden“ (Meyer et al. 2021).

6 Gefährdungsbeurteilungen beschreiben den Prozess der systematischen Ermittlung und Bewertung aller relevanten Gefährdungen, denen die Beschäftigten im Zuge ihrer beruflichen Tätigkeit ausgesetzt sind. Im Rahmen des Prozesses kommen die Ableitung und Umsetzung aller zum Schutz der Sicherheit und der Gesundheit erforderlichen Maßnahmen hinzu, die anschließend hinsichtlich ihrer Wirksamkeit überprüft werden müssen (vgl. §§ 5, 6 Arbeitsschutzgesetz und der Arbeitsstättenverordnung, § 3 Betriebssicherheitsverordnung, § 6 Gefahrstoffverordnung, §§ 89, 90 Betriebsverfassungsgesetz). Dabei müssen die Arbeitnehmer_innen gemäß EU-Richtlinie des Rates der Europäischen Gemeinschaft (89/391/EWG) angehört werden (Artikel 11).

7 Besonders stark zugenommen hat aus Sicht der Beschäftigten das Armutsrisiko in den Handlungsfeldern Sozia-le Arbeit mit Frauen (100 %), in Beratungsstellen (88,9 %), im Justizwesen (85,7 %), mit Menschen in prekären Lebenslagen (85,3 %) sowie im Bereich der migrationsbezogenen Tätigkeiten (80 %).

8 Besonders geringe Möglichkeiten zur digitalen Kontaktaufnahme mit den Beschäftigten der Sozialen Arbeit haben Adressat_innen in den Handlungsfeldern der Sozialen Arbeit mit Menschen in prekären Lebenslagen (74,7 %), mit älteren Menschen (68 %) oder dem Justizwesen (58,6 %).

9 „Zunächst haben drei Viertel der Teilnehmenden der Befragung (n = 1849) angegeben, in den letzten sieben Tagen (zum Zeitpunkt der Befragung) Kontakt zu den Adressat_innen gehabt zu haben; ein Viertel hat dies verneint“ (Buschle und Meyer 2020, S. 163).

10 Hier „gaben 19 % der Befragten an, dass in ihrer Einrichtung Hilfeprozesse früher als üblich beendet würden (*n* = 1561)“ (Meyer und Buschle 2020, S. 15).
